# Role of Meteorological Parameters in the Diurnal and Seasonal Variation of NO_2_ in a Romanian Urban Environment

**DOI:** 10.3390/ijerph17176228

**Published:** 2020-08-27

**Authors:** Mirela Voiculescu, Daniel-Eduard Constantin, Simona Condurache-Bota, Valentina Călmuc, Adrian Roșu, Carmelia Mariana Dragomir Bălănică

**Affiliations:** Faculty of Sciences and Environment, European Center of Excellence for the Environment, “Dunărea de Jos” University of Galați, 800008 Galați, Romania; Mirela.Voiculescu@ugal.ro (M.V.); scondurache@ugal.ro (S.C.-B.); valentina.calmuc@ugal.ro (V.C.); adrian.rosu@ugal.ro (A.R.); carmelia.dragomir@ugal.ro (C.M.D.B.)

**Keywords:** nitrogen dioxide, in situ urban concentrations, meteorological measurements, NO_2_ variation, partial correlation

## Abstract

The main purpose of this study was to investigate whether meteorological parameters (temperature, relative humidity, direct radiation) play an important role in modifying the NO_2_ concentration in an urban environment. The diurnal and seasonal variation recorded at a NO_2_ traffic station was analyzed, based on data collected in situ in a Romanian city, Braila (45.26° N, 27.95° E), during 2009–2014. The NO_2_ atmospheric content close to the ground had, in general, a summer minimum and a late autumn/winter maximum for most years. Two diurnal peaks were observed, regardless of the season, which were more evident during cold months. Traffic is an important contributor to the NO_2_ atmospheric pollution during daytime hours. The variability of in situ measurements of NO_2_ concentration compared relatively well with space-based observations of the NO_2_ vertical column by the Ozone Monitoring Instrument (OMI) satellite for most of the period under scrutiny. Data for daytime and nighttime (when the traffic is reduced) were analyzed separately, in the attempt to isolate meteorological effects. Meteorological parameters are not fully independent and we used partial correlation analysis to check whether the relationships with one parameter may be induced by another. The correlation between NO_2_ and temperature was not coherent. Relative humidity and solar radiation seemed to play a role in shaping the NO_2_ concentration, regardless of the time of day, and these relationships were only partially interconnected.

## 1. Introduction

Monitoring atmospheric pollution and the possibility to predict its evolution are of high interest. One major atmospheric pollutant is nitrogen dioxide (NO_2_), which may cause many health problems [1]. The NO_2_ gas has a reddish-brown color, is nonflammable, and has a detectable, pungent odor, perceptible from concentrations of approximately 190 μg/m^3^ [2]. Nitrogen dioxide reacts with the hydroxyl radical (-OH) in the atmosphere, forming the highly-corrosive nitric acid, but it can also form toxic organic nitrates. Nitrogen oxides known as NO_x_ (i.e., NO + NO_2_) are involved in the formation of tropospheric ozone and smog, mediated by light through photolysis. Due to the significant environmental impact, the NO_x_ compounds are strictly monitored and legislative limits were set at EU and national levels [2]. Part of the NO_2_ molecules in the atmosphere are of primary nature (i.e., directly emitted), while most NO_2_ results from nitrogen monoxide (NO) [1,3]. The latter is produced via natural and anthropogenic processes. About one-fifth of NO_x_ is released in the atmosphere during thunderstorms, through lightning, in the upper troposphere [3]. Other natural NO_x_ sources include volcanic activity emissions, bacteria decay, soil processes and stratospheric sources [1], while the rest is the result of fossil fuel combustion processes: fires, transportation, industrial—such as power generation, nitric acid manufacture, welding processes, the use of explosives and iron and steel industry and refineries [1,2]. According to [4], about 65% of NO_x_ emissions are of anthropogenic origin. The NO_2_ reaction with the atmospheric hydroxyl radical (-OH), and the NO reaction with hydroperoxyl (HO_2_), followed by the formation of nitric acid and its wet deposition are important NO sinks (almost 60%), according to [4]. The contribution of road traffic to nitrogen oxides (NO_x_) emissions has been described by various studies [5,6,7,8,9,10,11]. Some authors assume that about half of the anthropogenic NO_x_ comes from traffic [12]. Although several measures of emission control were implemented by various European Commission (EC) regulations, emissions of nitrogen oxides from road traffic have not significantly decreased across Europe; sometimes, NO_2_ concentrations have even increased [10,11]. Some recent papers showed that the real reduction of nitrogen oxides and other pollutants following Euro 5 and 6a/b introduction was not as important as expected [10].

The concentration of NO_2_ (and, in general, of any atmospheric pollutants) in an urban area and its variation are influenced by the industrialization level, the number of inhabitants and traffic density, but also by the topography of the area and meteorological parameters [5,9,11]. It is expected that meteorological factors would affect both natural and anthropogenic emissions of NO_2_ (e.g., the reduced solar radiation during wintertime will impact the rate of NO_2_ photochemical reactions and the reduced temperatures during the cold season will increase NO_2_ emissions associated with heating). Moreover, meteorological parameters are interlinked in various ways: the relative humidity decreases with increasing temperature, the effective and saturation air vapor pressures increase with atmospheric temperature, etc. Thus, it is difficult to assess the effect of each separate meteorological factor on NO_2_ pollution. None of these influences were yet quantified in a reliable way and a clear relationship is far from being established. Studies of the link between meteorological factors and atmospheric pollutants, in general, or NO_2_, in particular, are rather scarce, inconsistent and strongly depend on local factors [5,13,14,15,16,17,18,19]. According to [13], the NO_2_ concentration is slightly higher at a lower relative humidity, whereas other authors found that the NO_2_ concentration correlates positively with the relative humidity in all seasons, especially during winter [20]. The dependence between NO_2_ and temperature was found to be weak, in general, except for two considerable positive correlations, which were obtained for July and December, and for which no particular explanation was found in [13]. Other studies found strong positive correlations between the NO_2_ concentration and temperature for all seasons [20]. A negative correlation between wind speed and NO_2_ concentration in all seasons except for summer was found by [19].

This paper presents a study of the diurnal, monthly, seasonal and interannual evolution of nitrogen dioxide concentration at an urban site in the southeast of Romania (Braila, 45.3° N, 27.9° E), which is influenced by road traffic mainly during the daytime. The role played by anthropogenic sources and the most relevant meteorological parameters that may affect the level of NO_2_ pollution at the local scale is investigated. A comparison between in situ measurements of the NO_2_ concentration near the ground and satellite measurements of NO_2_ vertical columns is also performed. The timeframe for both in situ and satellite data is 2009–2014.

## 2. Data and Methods/Experimental

### 2.1. Data

The experimental data used in this study are from the automated monitoring air quality station called “BRAILA 1”, denoted as “BR1”, which is a traffic type station, one of the four available stations in Braila town, which are part of the National Network for Monitoring Air Quality (RNMCA, The Agency of Environmental Protection Braila: http://apmbr.anpm.ro/). The county of Braila, situated on the right shore of the Danube River, is considered to be the 11th Romanian city by the number of inhabitants. The number of vehicles in Braila has increased during the last few years, which led to a rather increased concentration of atmospheric pollutants. BR1 is a traffic station; it is located on Calea Galaţi No. 53 (The Agency of Environmental Protection Braila: http://apmbr.anpm.ro/) and monitors on a continuous basis the pollution levels generated mainly by traffic emissions, with medium and high flows, from the neighboring streets. Calea Galati Street is one of the busiest traffic streets in Braila. The width of the street is 16 m and it is covered with an asphalt carpet. The area around the air quality monitoring station has apartment blocks with four floors and some green spaces. The air quality monitoring station is located approximately 20 m from Calea Galati Street and 15 m from the apartment buildings. The surface is flat, characteristic of a plain area. The sources of pollution in the area consist mostly of road traffic and domestic heating.

The hourly atmospheric concentrations of NO_2_ are determined in situ, by using the chemiluminescence technique [8,21]. This method implies the reduction of NO_2_ to NO in the presence of a Molybdenum surface, at a temperature of roughly 310 °C. The resulted NO reacts with ozone (O_3_), leading to the formation of fluorescent NO_2_, whose emission is detected by a sensor [4,21]. Data between 2009 and 2014 were used in this study. Besides, concentrations of NO_2,_ meteorological parameters were also recorded.

Satellite measurements were provided by the Ozone Monitoring Instrument (OMI) onboard the Earth Observing System Aura satellite [22,23]. The OMI measures several important pollutants, such as O_3_, NO_2_, SO_2_, and aerosols, with a spatial resolution of 13 km × 24 km, i.e., at a near urban scale resolution, https://aura.gsfc.nasa.gov/omi.html [24]. OMI is a remote sensing space instrument onboard the Aura satellite that provides a raw NO_2_ product called DSCD (Differential Slant Column Density), which is retrieved using Differential Optical Absorption Spectroscopy (DOAS) in the 405–465 nm range. Satellite measurements provide valuable data about atmospheric pollutants, including NO_2_ [25,26,27,28,29,30,31,32] at a large scale. The most refined product of OMI is the tropospheric Vertical Column Density (VCD), which is the result of a near real-time retrieval algorithm that gives a 0.7 × 10^15^ molec./cm^2^ uncertainty for each individual pixel [26]. In our study, we have used level 3 data, which are a conversion of OMI 13 × 24 km^2^ data to a 0.25° × 0.25° resolution [33]. Such data collected between 2005 and 2015 by OMI confirmed a reduction in NO_2_ concentration over Northern China, Eastern Europe, and USA, while an increase in NO_2_ concentration was detected over the Persian Gulf and India [30].

### 2.2. Methods

Meteorological parameters recorded at an hourly rate were used, in order to verify to what extent they are relevant for modulating the NO_x_ variability by means of correlation analysis. Correlations/anticorrelations (i.e., positive/negative correlation coefficients) between two variables are not the decisive proof for direct cause–effect relationships; however, they suggest that a link may exist if a physical mechanism supports it. When two variables are not correlated, the first thought is that there is no link between the two variables, which, however, may not always be true. Moreover, meteorological parameters (temperature, humidity, solar radiation) are not independent (e.g., solar radiation at the ground is a measure of the cloud cover, which is linked to relative humidity but also to temperature). Consequently, the correlation analysis was refined and a partial correlation analysis was used [34,35], complementary to the bivariate correlation. Such an analysis is useful when the relationship between two variables, e.g., *X* and *Z*, measured by the bivariate correlation coefficient, *C_XZ_*, may be induced or suppressed by a third (intervening) variable, e.g., *Y*, which affects both variables *X* and *Z*. The partial correlation *P_X(Y)Z_* corresponds to the link between *X* and *Z* when *Y* is constant. To be more specific, in this particular case the main variables, *X* and *Z*, were the NO_2_ concentration and one meteorological factor (e.g., temperature) and the intervening variable, *Y*, was another meteorological parameter (e.g., radiation). The radiation (*Y*) may affect both the NO_2_ concentration (*X*) and the temperature (*Z*). The difference *D_X(Y)Z_* = *P_X(Y)Z_* − *C_XZ_* between the partial correlation coefficient, *P_X(Y)Z_,* and the direct correlation coefficient, *C_XZ_,* measures the degree of intervention for *Y*. If *P_X(Y)Z_* is smaller than *C_XZ_*, i.e., the difference and the direct coefficient have opposite signs and *C_XZ_ ×D_X(Y)Z_* < 0, then *Y* is responsible for part of the correlation, or it is said that Y “intervenes”. If the two coefficients, *C* and *D*, have the same sign, i.e., *C_XZ_ × D_X(Y)Z_* > 0, then the correlation is real and *Y* even suppresses, partially, the correlation. If the partial and direct coefficients are equal, i.e., *D* = 0, then the *Y* variable does not intervene. Table 1 summarizes possible results and interpretations for various combinations of direct and partial correlation coefficients.

The correlation analysis has been applied to standardized data (by their standard deviation) in order to identify possible links between NO_2_ concentration in the atmosphere and the variations of several meteorological parameters.

## 3. Results and Discussion

### 3.1. Diurnal and Seasonal Variation

**Figure 1:**
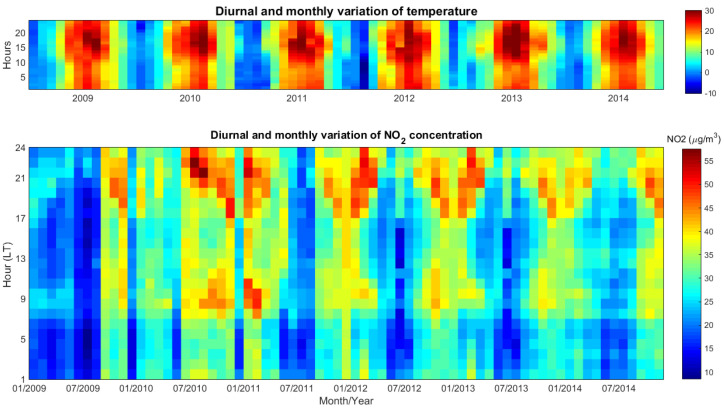
Diurnal and seasonal variation of NO_2_ concentration (**bottom**) and of temperature (**top**) during 2009–2014 (monthly means).

Figure 1 shows the diurnal and seasonal variation of NO_2_ concentration (top) and temperature (bottom) during 2009–2014. The hourly averaged NO_2_ concentration varied between 14 µg/m^3^ (July 2009) and 41 µg/m^3^ (January 2011). These values are smaller than those measured by traffic stations in Bucharest [28].

The NO_2_ content varied with year; it was low in 2009 and in the first part of 2010, reached a maximum in the winter between 2010 and 2011, and then slightly decreased towards the end of the interval. The period is too short for assessing whether a trend does exist.

The seasonal variation of NO_2_ was evident; NO_2_ concentrations were clearly higher during winter than during summer. The seasonal variation may be explained by: (1) the strong variation of the anthropogenic sources contributing to NO_2_ emissions, i.e., traffic and combustion, but also by (2) a longer lifetime of the NO_2_ during winter, taking into account that the NO_2_ lifetime and its concentration in the atmosphere are affected by the seasonal variation of the photochemical activity [7,8,26,31], which is reduced during winter. Additionally, during winter, the soil is cold, and thus the nearby air is heavy so that the emitted pollutants may stay close to the ground longer [2]. Another explanation for the seasonal NO_x_ variation relates to the variation of the boundary layer height with the season, which influences the wind pattern that, in turn, has a very important role in pollutant dispersion [7].

Two diurnal peaks of the NO_2_ concentration could be observed, centered around 09:00 Local Time (LT) and 20:00 LT, which were more evident during cold months. The diurnal peaks are in agreement with previous findings [8] and are associated with peaks in traffic [7]. Morning peaks, observed around 9:00, were smaller than the evening peaks. The NO_2_ concentration increased during the afternoon-evening period, between 17:00 and 24:00, with maxima progressing towards earlier time during winter months. This was seen in all seasons except summer. In December, these diurnal maxima were smaller, for all years, which can be linked to the reduced traffic and industrial activity due to holidays.

**Figure 2:**
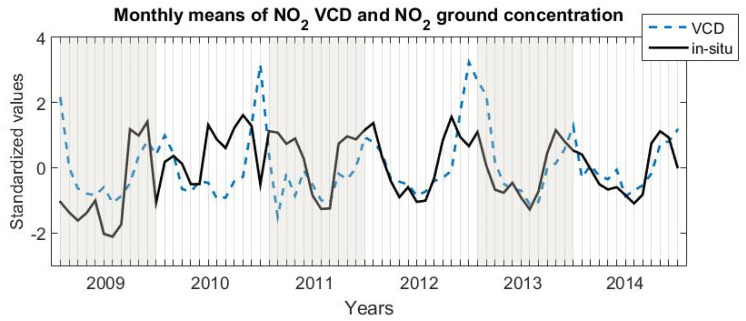
Variation of standardized monthly averages of tropospheric NO_2_ (VCD measured by OMI, blue, dash) and of NO_2_ ground concentration measured in situ at 11:00 LT (black, solid) starting with January 2009 until December 2014.

Figure 2 shows the variation of the tropospheric NO_2_ VCD (Vertical Column Density) over Braila, derived from OMI, together with in situ measurements at 11:00 LT, which is the approximate hour when the satellite overpasses the city. The tropospheric NO_2_ VCD varied between 0.8 × 10^15^ molec./cm^2^ for February 2011 and 4.7 × 10^15^ molec./cm^2^ for December 2012. Note that the comparison in Figure 2 is strictly qualitative, since OMI measures the number of NO_2_ molecules in a column over a grid of 0.25° [33], while in situ instruments measure the volumetric concentration near the ground; thus a direct quantitative comparison makes no sense. The annual NO_2_ VCD ranges between 1.73 and 2.23 × 10^15^ molec./cm^2^. Previous studies have shown that OMI measurements give reliable results about NO_2_ emissions of anthropogenic sources at a large scale in cities [26,32,36], above large industrial platforms (located away from cities) [25,37] and given by vehicle emissions and also by residential emissions [38]. However, other studies have shown that satellite observations do not correctly evaluate the NO_2_ pollution caused by traffic or by other point sources [27,28].

Figure 2 shows that the two time series agreed relatively well except for a period between the winter of 2009–2010 and the summer of 2011. The correlation coefficient between the two series was close to 0.40 (after values in December 2010 were removed) and did not change when NO_2_ concentrations measured earlier, between 08:00 and 11:00 LT, were considered for the mean.

The seasonal variation of the tropospheric VCD was more regular: it was small during warm months and high during cold months. In 2010, the two time series did not coincide, which is mainly due to a peculiar behavior of the in situ measurements that showed an unexpected wide maximum during summer and autumn, and a minimum in December. The OMI instrument “sees” a large surface area (312 km^2^), and thus integrates the emissions of many sources. Consequently, it cannot accurately measure the local variability, captured by the in situ measurements. This opposing variation suggests that a punctual, temporary, source of NO_2_ was added in the second part of 2010 close to the measuring station.

**Figure 3:**
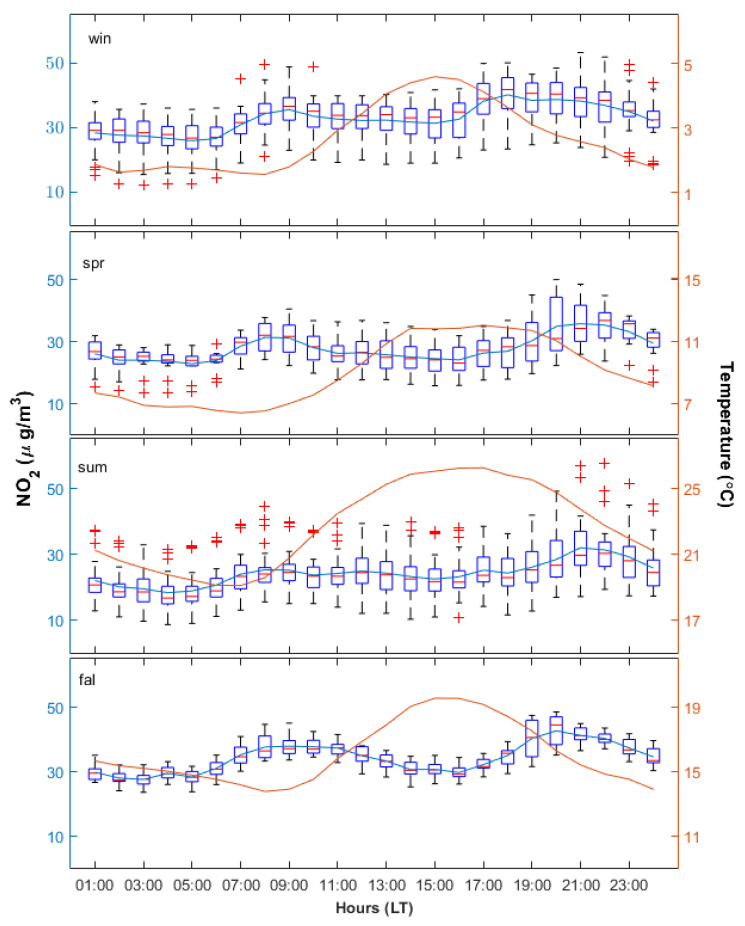
Diurnal variation of NO_2_ concentration (boxplots, left axis) for each season (top–down: spring, summer, fall and winter) for the entire interval, 2009–2014. See text for definition of seasons. The average temperature is superimposed (orange line, right axis).

Figure 3 shows the average diurnal variation of the NO_2_ concentration during each season for the entire period. Seasons are defined here in a slightly different manner than usual: spring and fall/autumn consist of two months each (March–April and September–October), while solstice seasons have four months: the summer covers the period between May and August, and the winter starts in November and ends in February. This definition of seasons is justified by the weather profile during the last 20 years in Braila County and in South-Eastern Romania, which shows a fast transition from winter to summer and then back to winter.

**Figure 4:**
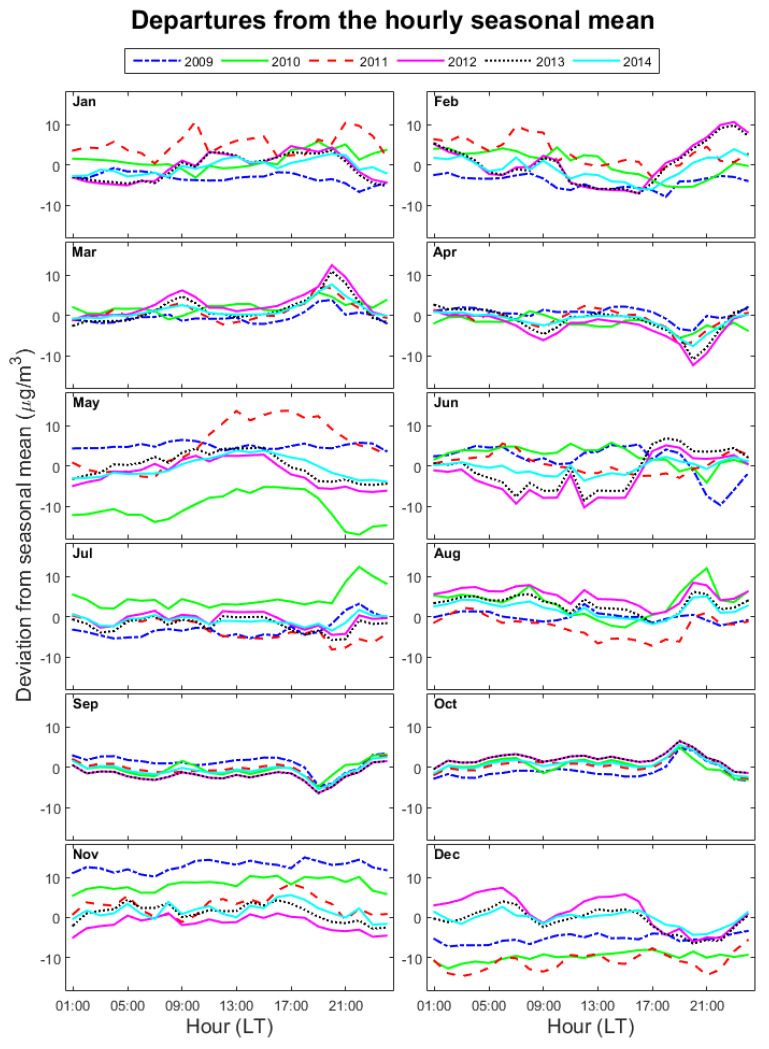
Departures of hourly NO_2_ concentration from the hourly seasonal means for each month, January to December. Each year is shown with different colors/line styles (see legend).

In general, the diurnal variation was relatively regular for fall, when a relatively small data spread was seen. A higher variability was observed in winter, spring and summer, when the data spread was larger. There were no outliers in the middle part of the day, when the traffic is slightly reduced compared to the morning and afternoon. The outliers during morning and afternoon may be associated with peaks or drop-offs of the traffic. The least regular season was summer, when the number of outliers was high regardless of the hour and their large majority lied in the upper part of the plot. This suggests that the NO_2_ loading was significantly higher than the average for a short period of time. This is confirmed by the next analysis (Figure 4). The explanation might relate to road construction works that resulted in deviation of the traffic. Starting with 2010, landslides occurred on streets near the measuring station. In 2011, the asphalt was removed and some excavations were done, in order to check the underground water and sewerage pipes. Subsequently, utility pipes were installed, the foundation was compacted and the traffic flow returned to normal values.

Apparently, the NO_2_ concentration and the temperature were anticorrelated, especially during daytime: high NO_2_ concentrations during morning corresponded to the lowest temperatures, while the afternoon reduction in NO_2_ coincided with the highest temperatures. However, this is just coincidental, since the daytime variation of NO_2_ is governed by traffic [8], whose peaks happen to occur at times when the temperature is lowest. Moreover, during nighttime, the NO_2_ concentration and the temperature seemed to be correlated.

### 3.2. Monthly Deviation of NO_2_ Concentration from the Seasonal Mean

Obviously, the diurnal variation of NO_2_ was not the same for each year. Figure 4 shows the difference between the hourly NO_2_ concentrations at ground level and the hourly seasonal mean during each month of each year. Hourly seasonal means are averages of hourly NO_2_ values measured during 2009 and 2014 over those months that define a season, i.e., November–February for winter, March–April for spring, May–August for summer and September–October for fall.

The analysis of the temporal variation of the aforementioned differences may be useful for identifying months or periods of the day when the NO_2_ loading departed from the expected variability, which in turn may help in finding the cause for the outliers seen in Figure 3. Values lying in the positive/negative part mean that the NO_2_ concentration were higher/lower than the average at that particular time.

A clear pattern could be observed for equinox seasons: the afternoon peak was higher during colder months (March and October) than during warmer months (April and September) for each year. This was not true for the morning peak or for solstice seasons. The month of May, which is part of the summer season, was the least regular; the NO_2_ content in 2010 was much lower, while in 2011 it was much higher than the average. The unusual increase during the second part of 2010, shown in Figure 2, is confirmed by the higher values seen in Figure 4 for July 2010 and, partially, for June 2010. The explanation might relate to the construction works previously described, which resulted in significant alterations of the traffic flow during 2010–2011. In general, the major contributor to the summer mean for all years came from the NO_2_ content in August, since the departures from the seasonal mean were positive for all years except 2011.

The NO_2_ content in winter also depended on month and year. November seemed to be the month with the highest contribution to the winter seasonal average of NO_2_ concentration for the first part of the interval (2009–2011). In December, the NO_2_ diurnal variability changed with the year: the NO_2_ content was lower during the first three years and higher afterwards. We assume that the explanation lies in a combination of meteorological conditions and important variations of traffic and industrial emissions during these particular years. During the analyzed period, the distribution of thermal energy and hot water in Braila municipality was mainly controlled by the heating operator SC “CET” SA. Starting with 2012, the lack of investments in modernizing the heating system affected the control of emissions: severe losses and the evolution of the methane gas tariff led to an increase of classical housing heating systems, whose impact is important especially during the cold season [39].

### 3.3. Correlation between NO_2_ Concentration and Meteorological Parameters

The effect of meteorological parameters on the variability of NO_2_ concentration for urban sites is, still, a conundrum. Table 2 shows some examples of correlations between NO_2_ and meteorological factors for different locations [13,14,15,16,17,18,19,20]. The correlation between temperature and humidity, on the one side, and NO_x_, on the other side, was positive, negative or insignificant. The correlation with the wind speed was more consistent, i.e., is negative for all sites, which is rather normal, since a stronger wind will disperse the NO_2_ at a local urban site. This is the reason for investigating whether meteorological parameters may be linked to the variability of the NO_2_ in a relatively small city, with an average level of pollution [31] and whether this relationship depends on seasons or on time of day.

Figure 2, Figure 3 and Figure 4 show that during about 07:00 and 21:00 LT, the NO_2_ variability was strongly influenced by traffic, which is also confirmed by [8,37,40,41,42]. The correlation coefficients were computed separately for the full 24 h time (black bars), for daytime (8–21 LT, red bars) and for the nighttime (22–7 LT, blue bars). The separation between daytime and nighttime was done because the influence of the road traffic should be lower during nighttime, and thus meteorological parameters may play a different role in modulating the NO_2_ concentration during the night. Obviously, this is not valid for radiation, which is absent during nighttime. However, one should keep in mind that the NO_2_ content is largely controlled by traffic, especially during the day, and this will definitely affect the correlation with meteorological parameters (or lack thereof). However, we assumed that the traffic pressure does not change for the analysis period.

**Figure 5:**
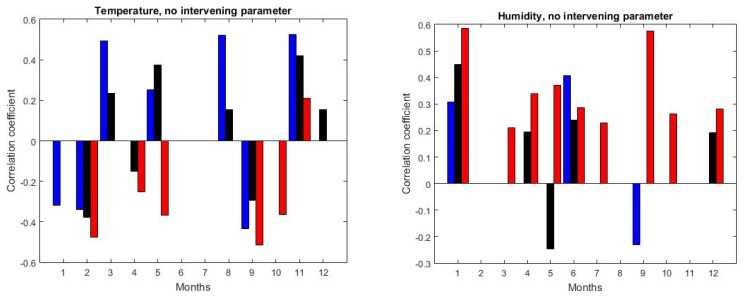
Full bivariate correlation of NO_2_ with the temperature (**left**) and humidity (**right**). Coefficients (>90% confidence level) are shown for 24 h by black bars, while red/blue bars correspond to day/night, respectively—see text for details.

Figure 5 shows the variation of the direct bivariate correlation between NO_2_ and temperature (left), and humidity (right) with time. Correlation coefficients calculated for the full day are shown by black bars, while for day (night) these are shown by red (blue) bars. Only coefficients that were significant at 90% are shown.

There was no clear NO_2_ dependence on temperature, since correlation coefficients were positive for March, May, August and November, while for February and September these were negative. During daytime most correlations were negative; however, this was already discussed as being artificially induced by rush-hour traffic. Correlations did not change from day to night, except for May, when the correlation changed from negative for the full-day to positive for the full day and night. The full 24 h correlation was rather small or insignificant for most months and changed from positive to negative during months that had similar meteorological characteristics, e.g., March (positive), April (negative) and May (positive). Unsurprisingly, NO_2_ and temperature were anticorrelated during the day, but this was already discussed as being mostly artificial. Significant correlation during the night was positive in March, May, August and November, and negative in January, February and September. All in all, temperature seemed to play no clear role in the variability of NO_2_.

Negative coefficients were found for the NO_2_–temperature dependence by [14,16,17,18], without a clear association with seasons. The NO_2_ lifetime is higher during winter; thus the “night” analysis may be less relevant during winter because of the lower concentrations of the N_2_O and N_2_O_5_ species. These species are key factors in the removal of NO_2_ during nighttime and in its transformation into nitric acid [43]. The amount of NO_y_ species is higher during warmer seasons when bacteria and agriculture activities are intensified [44]. However, [15,19] found that an increase in temperature would be followed by an increase in NO_x_.

The correlation of the NO_2_ concentration with the humidity, when significant, was positive for most months. The only exception was May, during which the NO_2_ variability departed significantly from the expected behavior (Figure 4). The correlation did not change from day to night except in September.

In the following the intervening effect on direct correlations is analyzed, to see whether the existing correlations were spuriously induced, especially for the link to humidity. The partial correlation was used to identify whether the effects of meteorological parameters (temperature, relative humidity and solar radiation) on NO_2_ concentration were interconnected. The main and intervening variables (described in Section 2) are shown in Table 3. The results are shown only for four out of the six possible combinations, because these are the most relevant for identifying possible intervening effects. The correlation between NO_2_ and temperature was inconsistent and when assessing the intervening effect of radiation on correlation with humidity one can infer that the intervening effect of humidity was similar.

**Figure 6:**
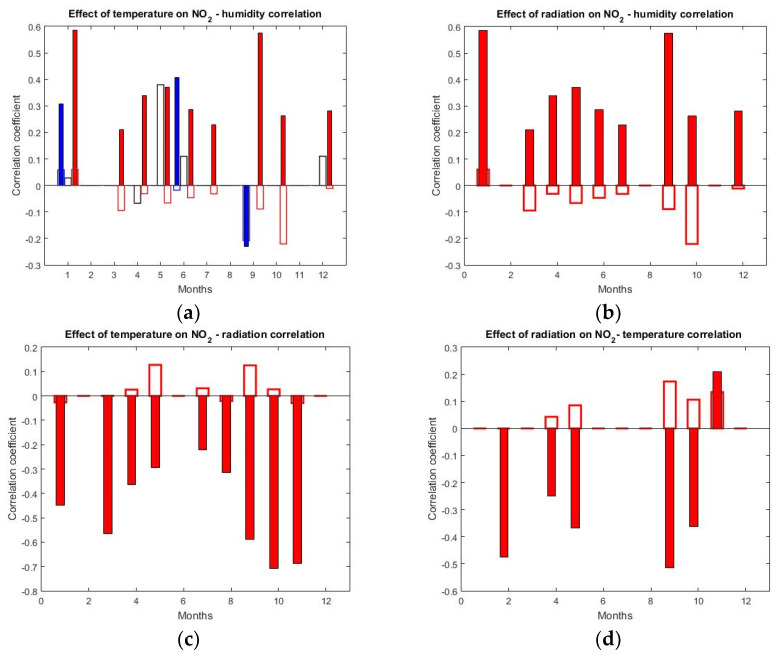
Partial correlation between NO_2_ concentration and meteorological parameters. Full bars describe the bivariate correlation and empty bars describe the difference (*D*) between the partial and bivariate correlation. Correlation coefficients for the whole day are shown in black, while red/blue correspond to day/night, respectively—see text for details. Coefficients are shown for the >90% confidence level. Partial correlation between **NO_2_ and humidity** is shown in (**a**) (*temperature* constant) and (**b**) (*radiation* constant). Partial correlation between **NO_2_ and radiation** is shown in (**c**) (temperature constant) and the effect of *radiation* on the correlation with **temperature** is in (**d**).

Figure 6 shows the results of the partial correlation analysis for the combinations in Table 3. Full bars describe the direct correlation, while empty bars stand for the differences between partial and direct coefficients. According to Table 1, when these two are opposite, the correlation is partially mediated by the intervening variable. When these both have the same sign, the correlation is real.

No consistent direct correlation between NO_2_ and humidity was found for the whole 24 h period (Figure 6a, full black bars are completely absent). Notably, *D_NO(t)H_* was rather high and positive for the whole 24 h day during May, June and December. This means that the temperature suppressed a potentially positive NO_x_–humidity correlation. During daytime, NO_2_ and humidity were positively correlated (red full bars) for a large part of the year, except for February, August and November. The differences between partial and direct correlation, *D_NO(t)H_*, albeit small, were of opposing signs for most cases. This suggests that the temperature was partially responsible for the NO_2_–humidity correlation.

The important role played by the humidity in the variation of NO_2_ is supported by Figure 6b, where the effect of radiation on the same correlation (NO_2_–humidity) is shown only for daytime. The solar radiation partially induced a positive correlation during some months, but the effect was not important, since all *D_NO(R)H_* were pretty small. Our results agree with [19] or [20], but contradict [13], who showed that NO_2_ was negatively correlated with relative humidity. They argued that NO_2_ concentrations are slightly higher at a lower relative humidity because the reactions between NO_2_ and OH are less frequent, and thus the NO_2_ persists more in the atmosphere. However, this was not confirmed by our results.

The NO_2_ concentration was negatively correlated with solar radiation during the entire year. A higher direct radiation implies, usually, a higher air temperature; thus the intervening effect of temperature and radiation was analyzed. Figure 6c shows that the temperature did not artificially induce the anticorrelation with solar radiation (small or absent empty bars), except for May and September, when the effect was, however, small. This holds for the intervening effect of solar radiation on the anticorrelation with temperature, (Figure 6d), which was also small.

Based on observations in [45], which showed that, for a site in India, O_3_ correlated negatively with both NO_x_ and the humidity during all seasons, we suggest that the positive correlation between NO_x_ and humidity may be an indirect result of the photochemical effect of solar radiation and humidity on ozone. Unfortunately, ozone measurements were not available to confirm this hypothesis. This also may partly explain the observed anticorrelation between NO_2_ and solar radiation. Increased solar radiation favors the production of O_3_, which, in turn, reduces the NO_2_ loading in the atmosphere [45].

In general, comparisons with other studies are not straightforward, since we are not aware of similar investigations of the intervening effect of meteorological parameters. Additionally, most studies did not consider monthly changes. Moreover, correlations between the NO_2_ concentration and the meteorological parameters should be different for different cities, since the anthropogenic landscape and microclimate change significantly from one urban location to another [10,31].

## 4. Conclusions

This paper describes the diurnal, monthly, seasonal and annual evolution of the NO_2_ concentration for 2009–2014, measured in situ by an urban traffic station in southeast Romania. The role that meteorological parameters might play in modulating the NO_2_ variability was investigated in an attempt to separate the anthropogenic effects (which are well-known) from the effect of the local microclimate.

As expected, the NO_2_ was higher during the cold season, except for one year, 2010, when summer NO_2_ levels were highest. This suggests that natural factors, such as the effect of temperature on the NO_2_ lifetime, are less important than the anthropogenic ones at urban sites. Some annual variation also existed, with low values at the beginning of the interval (2009–2010), most likely caused by a severe reduction of industrial activity.The summer minima and winter maxima have both anthropogenic and natural causes and the departure from these may relate to temporary changes of the local traffic and/or construction activities. The NO_2_ diurnal variability was clearly shaped by the local transport: two diurnal peaks were observed, one around 8–9 LT and another one around 20–21 LT, and both were associated with increased road traffic, confirming previous observations at other urban sites. The afternoon peak was higher during the colder months (March and October) than during the warmer months (April and September) for each year. An irregular diurnal variation of the NO_2_ concentration was seen in May and December. The most consistent season was autumn, with a relatively similar diurnal variation in all years.

Additionally, we found that over Braila, space observations of OMI followed the in situ observations during most of the selected interval (R = 0.4).

The analysis of the correlations between the NO_2_ concentration and temperature, relative humidity and radiation has shown that the association with temperature is the least relevant. The correlation changed from positive to negative throughout the year without a clear pattern. Obviously, the contribution of traffic cannot be disregarded and may mask or suppress the impact of temperature variations on the NO_2_ concentration. Our assumption that during the night, the situation may change due to traffic disappearance, was not confirmed. The correlations with the humidity and radiation, on the other hand, were notably consistent: the NO_2_ concentration correlated with the relative humidity and was anticorrelated with radiation for almost the entire year. Moreover, most of these relationships were real and the intervening effect of the other meteorological parameters was small.

Our results showed that finding a link between meteorological parameters and NO_2_ variability for an urban site is a difficult task. Attempts to predict the NO_2_ behavior based on meteorological data, even combined with traffic flux data, cannot be very successful at the local or regional scale or on a short-term basis, since landscape, infrastructure, traffic, local activities, and population clearly affect the NO_2_ concentration. However, this may be useful for assessing trends or long-tern variability at a large scale, since these are averaging over a large area, thus reducing local and short-term contributions.

## Figures and Tables

**Table 1:** Type of correlation for various results of the partial correlation analysis.

*C* (Bivariate/Direct Correlation)	*D* = *P* − *C*	*C × D*	Correlation between *X* and *Z*	Role of the Intervening Variable (*Y*)
>0	0	0	real (positive) correlation	none
>0	<0	<0	partially spurious	partially responsible for the correlation
>0	>0	>0	partially suppressed	partially masks the real correlation
0	any	0	fully suppressed	fully masks the real correlation
<0	0	0	real (negative) correlation	none
<0	<0	>0	partially suppressed	partially masks the real correlation
<0	>0	<0	partially spurious	partially responsible for the correlation
any	=*C*	>0	fully spurious	fully responsible for the correlation

**Table 2:** Correlations between NO_2_ and meteorological parameters for various locations.

Location	Temperature	Humidity	Wind Speed	Seasons	Reference
Egypt, Cairo	Insignificant	Negative	Negative	-	[13]
India, Surat	Insignificant	-	Negative	Summer Autumn	[14]
India, Jabalpur	Negative	Negative	-	-	[17]
Saudi Arabia, Makkah	Insignificant	Negative	Negative	-	[18]
Saudi Arabia, Dhahran	Negative	Positive	Negative	Summer	[20]
China, Beijing	Negative	Positive	Negative	-	[19]
China, Shanghai	Negative	Negative	Negative	-	[19]
China, Guangzhou	Positive	Negative	Negative	-	[19]
Malaysia Kuala Lumpur	Positive	Insignificant	Negative	-	[15]
Iran, Isfahan	Negative	Negative	Negative	-	[16]

**Table 3:** Meteorological parameters as main/intervening variables, for the partial correlation analysis.

Variable 1 (*X*)	Variable 2 (*Z*)	Intervening Variable (*Y*)	Plot in Figure 6
NO_2_	relative humidity	temperature	a
NO_2_	relative humidity	radiation	b
NO_2_	radiation	temperature	c
NO_2_	temperature	radiation	d
